# Evaluation of potential anti-metastatic and antioxidative abilities of natural peptides derived from *Tecoma stans* (L.) Juss. ex Kunth in A549 cells

**DOI:** 10.7717/peerj.13693

**Published:** 2022-07-06

**Authors:** Sucheewin Krobthong, Yodying Yingchutrakul, Wattanapong Sittisaree, Tatpong Tulyananda, Pawitrabhorn Samutrtai, Kiattawee Choowongkomon, Udom Lao-On

**Affiliations:** 1 Center for Neuroscience, Faculty of Science, Mahidol University, Bangkok, Thailand; 2 Interdisciplinary Graduate Program in Genetic Engineering, Kasetsart University, Bangkok, Thailand; 3 National Omics Center, National Science and Technology Development Agency (NSTDA), Pathum Thani, Thailand; 4 Merck Life Science Thailand, Merck Ltd., Bangkok, Thailand; 5 School of Bioinnovation and Bio-Based Product Intelligence, Faculty of Science, Mahidol University, Bangkok, Thailand; 6 Department of Pharmaceutical Sciences, Faculty of Pharmacy, Chiang Mai University, Chiang Mai, Thailand; 7 Department of Biochemistry, Faculty of Science, Kasetsart University, Bangkok, Thailand; 8 Department of Medical Technology, School of Allied Health Sciences, Walailak University, Nakhon Si Thammarat, Thailand; 9 Hematology and Transfusion Science Research Center (HTSRC), Walailak University, Nakhon Si Thammarat, Thailand

**Keywords:** Anti-metastasis, Antioxidant, Medicinal plant, Natural peptide, Proteomics

## Abstract

**Background:**

*Tecoma stans* (L.) Juss. ex Kunth is a well-known medicinal plant found in tropical and subtropical regions. It contains a broad range of bioactive compounds that exhibit many biological effects, including antidiabetic, antibacterial, and antioxidative activities. However, the effect of natural peptides from *T. stans* against cancer progression and free radical production is unknown. This study aims to evaluate the cytotoxic, anti-metastatic, and antioxidative activities of natural peptides from *T. stans* on A549 cells.

**Methods:**

The natural peptides were extracted from the flower of *T. stans* using the pressurized hot water extraction (PHWE) method, followed by size exclusion chromatography and solid-phase extraction-C18. The cytotoxic and anti-metastatic effects of natural peptides were evaluated using MTT and transwell chamber assays, respectively. The free radical scavenging activity of natural peptides was determined using ABTS, DPPH, and FRAP assays. The cells were pretreated with the IC_50_ dosage of natural peptides and stimulated with LPS before analyzing intracellular reactive oxygen species (ROS) and proteomics.

**Results:**

Natural peptides induced cell toxicity at a concentration of less than 1 ng/ml and markedly reduced cell motility of A549 cells. The cells had a migration rate of less than 10% and lost their invasion ability in the treatment condition. In addition, natural peptides showed free radical scavenging activity similar to standard antioxidants and significantly decreased intracellular ROS in the LPS-induced cells. Proteomic analysis revealed 1,604 differentially expressed proteins. The self-organizing tree algorithm (SOTA) clustered the protein abundances into eleven groups. The volcano plot revealed that the cancer-promoting proteins (NCBP2, AMD, MER34, ENC1, and COA4) were down-regulated, while the secretory glycoprotein (A1BG) and ROS-reducing protein (ASB6) were up-regulated in the treatment group.

**Conclusion:**

The anti-proliferative and anti-metastatic activities of natural peptides may be attributed to the suppression of several cancer-promoting proteins. In contrast, their antioxidative activity may result from the up-regulation of ROS-reducing protein. This finding suggests that natural peptides from *T. stans* are viable for being the new potential anti-cancer and antioxidative agents.

## Introduction

Lung cancer is the most common cancer in males and the third cancer in females. It is the leading cause of cancer-related death worldwide (Sung et al., 2021). There are two major types of lung cancer, small-cell lung cancer (SCLC) and non-small-cell lung cancer (NSCLC) (Travis et al., 2015). NSCLC accounts for 85% of all lung cancer, and it is divided into three subtypes: squamous cell carcinoma, adenocarcinoma, and large-cell carcinoma (Zheng, 2016). Lung cancers usually have a poor prognosis in which over half of patients die within 1 year of diagnosis, and the 5-year survival is less than 18% (Zappa & Mousa, 2016). The high mortality rate of lung cancer is mostly observed in patients with the advanced or metastatic stage at the time of diagnosis (Berghmans, Paesmans & Sculier, 2011). Metastasis is the leading cause of cancer-related death, with approximately 90% of lung cancer patients (Chanvorachote et al., 2016). Lung cancers have certain preferential sites for metastasis, such as bones, brain, liver, and adrenal glands (Popper, 2016). Colonization of metastasized lung cancer cells at the secondary organs is attributed to more patients’ deaths compared to primary lung cancer. Metastasized lung cancer cells are also associated with chemotherapy resistance leading to therapeutic failure and recurrence (Schiller et al., 2002). Thus, exploring the potential anti-metastatic agents is important for advanced lung cancer treatment.

Free radicals are derived from either normal cellular metabolism or exposure to external factors such as air pollutants, ultraviolet radiations, and industrial chemicals. They are unstable, short-lived, and highly reactive. The high amount of free radicals or ROS has adverse effects as they can easily damage all kinds of macromolecules, including proteins, genetic materials, and lipids (Dröge, 2002). However, they can be eliminated or balanced by antioxidants. An antioxidant is a molecule that can neutralize free radicals by stabilizing them before a cellular attack. The principal antioxidants are the cellular enzymes. The crucial enzymatic antioxidants include superoxide dismutase (SOD), catalase (CAT), and glutathione peroxidase (GPx) (Ighodaro & Akinloye, 2018). The SOD catalyzes superoxide anion (O_2_^−^) to hydrogen peroxide (H_2_O_2_), which is then converted to water by either catalase or glutathione peroxidase (Lee & Kang, 2013). Meanwhile, general antioxidants are the agents that can neutralize or scavenge free radicals by donating electrons to them (Das & Roychoudhury, 2014). The most familiar antioxidative agents are ascorbic acid, reduced glutathione, α-tocopherol, carotenoids, flavonoids, and proline. Antioxidants can be considered a health-promoting agents because of their free radical scavenging activity. Although there are synthetic and artificial antioxidative agents such as tertiary butyl-hydroquinone, butylated hydroxyanisole, and butylated hydroxytoluene, their undesirable effects limit their use (Huang et al., 2015). Therefore, searching for alternative antioxidants that are natural, safe, and produced using green approaches has gained much attention.

*Tecoma stans* (L.) Juss. ex Kunth (Bignoniaceae) is a medicinal ornamental plant. Bioactive compounds from *T. stans* such as alkaloids, flavonoids, saponins, and tannins have shown antidiabetic, antibacterial, antifungal, and anti-cancer activities (Aguilar-Santamaría et al., 2009; Alonso-Castro et al., 2010; Salem et al., 2013). Several compounds (phenylethanoid and monoterpene alkaloid) from the flowers of the *T. stans* plant have antioxidative activity and anti-proliferative effects against cancer cell lines (Marzouk et al., 2006). The peptides, which are primary metabolites from natural sources, are economically viable and have several biological effects on the cells (Hsiao et al., 2014; Krobthong et al., 2021). The anti-cancer peptides are considered to be the novel potent anti-cancer agents as they can broadly inhibit tumor progressions such as proliferation, migration, and angiogenesis (Xie, Liu & Yang, 2020). Meanwhile, antioxidative peptides have drawn the attention of health-conscious consumers and scientists because ROS is involved in many health and medical care (Tadesse & Emire, 2020). According to the abundance of raw material and economic value, natural peptides from *T. stans* gained much interest in contributing anti-cancer and antioxidative peptides. However, the inhibition of cell viability, metastasis, and oxidative stress in A549 cells by natural peptides from *T. stans* has not been reported. Therefore, the present study aimed to investigate the inhibitory effect of natural peptides from *T. stans* on cell viability, migration, invasion, and intracellular ROS production in A549 cells.

## Materials and Methods

### Materials

The chemicals, materials, and equipment follow: Flower of *Tecoma stans* (L.) Juss. ex Kunth was collected in April 2021 in Pathuntani, Thailand (5.7800° N, 101.0372° E). For quality control (QC), the samples were collected in three biological samplings and conducted in independent extraction processes. An ultracentrifugal mill model ZM-200 was purchased from the Retsch Co. (Haan, Germany). A rotatory evaporator (model: R300) was purchased from Buchi Co. (Flawil, Switzerland). Synergy H1 UV-Vis spectrometer microplate reader was purchased from BioTek Co. (Winooski, VT, USA). Vivaspin^®^ 20 Molecular weights cut off (MWCO) 3 kDa, dithiothreitol (DTT), and iodoacetamide (IAA) were purchased from GE Healthcare Co. (Amersham, UK).

Human non-small cell lung cancer cell line, A549 (ATCC: CLL-185); human hepatocarcinoma cell line, HepG2 (ATCC: HB-8065); human cervical adenocarcinoma cell line, HeLa (ATCC: CCL-2); human melanoma cell line, SK-MEL-28 (ATCC: HTB-72) and human breast cancer cell line, MCF-7 (ATCC: HTB-22) were purchased from the American Type Culture Collection (Manassas, VA, USA). Immortalized human keratinocyte cell line, HaCat (CLS: 300493) was purchased from Cell Lines Service. The biochemical reagents, including Dulbecco’s modified Eagle’s medium (DMEM), fetal bovine serum (FBS), and penicillin-streptomycin, were purchased from Gibco (Grand Island, NY, USA). The dimethyl sulfoxide (DMSO), methylthiazolyldiphenyl-tetrazolium bromide (MTT), 1,1-diphenyl-2-picrylhydrazyl (DPPH), ascorbic acid (As), gallic acid (GA), 2,2′-azino-bis (3-ethylbenzothiazoline-6-sulfonic acid) (ABTS), K_2_S_2_O_8_, FeCl_3_, 2,4,6-tris(2-pyridyl)-s-triazine (TPTZ), and FeSO_4_ were obtained from Sigma Aldrich Co. (St. Louis, MO, USA). A cellular ROS detection kit, 2′–7′ dichlorofluorescein diacetate (DCFH-DA), was purchased from Abcam Co. (Cambridge, UK). The transwell insert (6.5-mm diameter polyvinylpyrrolidone-free polycarbonate filter of 8-µm pore size) and Matrigel were purchased from Corning Inc. (Corning, NY, USA).

Trypsin, used for sequencing, was purchased from Promega Co. (Madison, WI, USA). Rapigest SF surfactant was purchased from Waters Co. (Milford, MA, USA). Halt protease inhibitor cocktail and benchtop centrifuge (Sorvall™ Legend™ Micro 17) were purchased from Thermo Scientific Co. (Waltham, MA, USA). Solvents for LC-MS/MS, including LC-MS waters and acetonitrile (ultra LC-MS), were purchased from J.T. Baker (Fisher Scientific, Loughborough, UK). All other biochemical reagents were purchased from Sigma Aldrich Co.

### Natural peptide enrichment and purification

Surface sterilization of the flower of *T. stans* was done using 0.1% Tween-80 solution and washed five times with deionized water at room temperature (25–26 °C). After sterilization, the *T. stans* was dried (10 g) using a hot-air oven at 40 °C for 2 days on an aluminum foil and then grounded into small particle size by the ultra-centrifugal mill at 8,000*g* for 3 s. The grounded *T. stans* was extracted using high pressure and temperature in deionized water (121 °C at 15 psi for 20 min) at 1:2 (w/v) ratio. The extracted *T. stans* was left to room temperature, then added absolute ethanol at 1:1 (v/v) ratio. The supernatant was filtrated through the MWCO 3 kDa. The natural peptides fraction was collected. The natural peptide enrichment and purification using solid-phase extraction (SPE) cartridges were conducted. The SPE cartridges were pre-conditioned using 20 ml acetonitrile and equilibrated with 15 ml deionized water. The flow-through from MWCO was loaded on the equilibrated SPE and eluted with 20% acetonitrile. An eluent was then evaporated under a vacuum using the rotatory evaporator. For investigating the extract quality using LC-MS/MS analysis, Orbitrap HF hybrid mass spectrometer combined with an UltiMate 3000 LC system was used as described previously (Treesubsuntorn et al., 2021). Briefly, the peptides were first desalted one-line on a reverse-phase C18 PepMap 100 trapping column before being resolved onto a C18 PepMap™ 100 capillary column with a 60 min gradient of CH_3_CN, 0.1% formic acid, at a flow rate of 300 nl/min. Peptides were analyzed by applying a data-dependent Top10 method consisting of a scan cycle initiated by a full scan of peptide ions in the Orbitrap analyzer, followed by high-energy collisional dissociation (NCE = 28) and MS/MS scans on the 15 most abundant precursor ions. Full scan mass spectra were acquired from *m/z* 200 to 2,000 with an AGC target set at 3 × 10^6^ ions and a resolution of 120k. MS/MS scan was initiated when the AGC target reached 10^5^ ions and a resolution of 30k. Ion selection was performed applying a dynamic exclusion window of 15 s. The peptide peak picking using PPD algorithm with signal/noise ratio >1 by an automatic peak detection tools within Thermo Scientific FreeStyle software. The natural peptides profile was illustrated in total ion counts, parent masses (MS), and daughter masses (MS/MS) profiles. The raw mass spectra were analyzed by PeakX studio 10.0 program. *De novo* peptide sequencing of the highest peptide ion intensity was performed with default parameters. LC-MS run was analyzed with no specific digestion enzyme. Mass error tolerance for MS and MS/MS was 10 ppm and 0.01 Da, respectively. CID-Fragmentation series (b, y, b-H_2_O, y-H_2_O, and y-NH_3_) were used to predict peptide sequence (Krobthong & Yingchutrakul, 2020). The acceptable *de novo* peptide sequences were achieved by filtering average local confidence to ≥95%.

### Cell viability assay

The cytotoxic effect of natural peptides from the flower of *T. stans* was determined by MTT assay. A549, HepG2, HeLa, SK-MEL-28, MCF-7, and HaCat cells were plated in a 96-well plate at a density of 1 × 10^4^ cells and incubated for 24 h. After incubation, the cells were treated with natural peptides from *T. stans* at different concentrations (2.0, 1.0, 0.5, 0.25, 0.125 and 0.0625 ng/ml for cancer cells and 25.0, 12.5, 6.25, 3.125, 1.563, 0.781 and 0.391 ng/ml for immortalized cells) or negative control (0.01% DMSO) for 24 h. After treatment, 0.5 mg/ml of MTT solution was added to each well, and the plate was further incubated for 2 h at 37 °C. The MTT assay medium was removed, and 100 µl of DMSO was added to each well. The cell cytotoxicity was measured using absorbance at 570 nm and transformed to the percentage of the cell viability. The inhibitory concentration (IC_50_) was analyzed using the non-linear sigmoidal model of GraphPad Prism 8.0.1 software (San Diego, CA, USA). Cell viability assay was performed with three independent experiments. The dosage of natural peptides from *T. stans* at the IC_50_ was used in the intracellular ROS evaluation and proteomic analysis.

### Migration and invasion assays

The effect of natural peptides from the flower of *T. stans* on cell migration and invasion was assayed by transwell chambers. A549 cells were seeded in a 24-well plate and treated with or without the lowest concentration (0.0625 ng/ml) of natural peptides for 24 h. After treatment, cells were harvested, and 1.0 × 10^5^ cells were suspended in 200 µl of the serum-free medium before plating into the upper chamber of transwell, which was uncoated (migration assay) or pre-coated (invasion assay) with 30 µg of Matrigel. Cells were allowed to migrate or invade through transwell to the lower chamber containing 750 µl of complete medium with 10% FBS, which was used as a chemoattractant at 37 °C for 24 h. After incubation, the cells in the upper surface of the membrane were removed with a cotton swab, and the chamber was washed twice with 1× PBS. The cells that had migrated across the membrane or invaded through the Matrigel were fixed with 3.7% (v/v) formaldehyde in 1× PBS for 20 min. The cells were stained with 0.5% crystal violet in 25% (v/v) methanol overnight, followed by two washes with tap water. Finally, the migrated or invaded cells on the undersurface of the membrane were counted and photographed under a microscope. The data are presented as the percentage of migration or invasion through the uncoated or pre-coated Matrigel membrane, respectively.

### *In vitro* radical scavenging assay of the natural peptides by using DPPH, ABTS and FRAP assays

The evaluation of the relative antioxidant capacity of natural peptides derived from the flower of *T. stans* was carried out using DPPH, ABTS and FRAP (Ferric reducing antioxidant power) assays, as mentioned previously (Krobthong et al., 2021). An antioxidant capacity was determined at the fixed concentration of 1 μg/μl of the natural peptides. For DPPH scavenging activity, the 10 µl of natural peptide solution was mixed with 190 µl of 0.2 mM DPPH in the absolute ethanol. The absorbance was measured at 517 nm. DPPH antioxidant efficiency used ascorbic acid (As) as the standard. The DPPH scavenging activity was expressed as the ascorbic acid equivalent antioxidant capacity (AsEAC).

For ABTS scavenging activity, ABTS radical solution (7 mM ABTS solution with 2.45 mM potassium persulfate) was diluted in 5 mM phosphate buffer saline solution, pH 7.2. The 10 µl of natural peptide solution was mixed with 190 µl ABTS solution. The experiment was incubated at room temperature in the dark for 15 min. The absorbance of the mixture was measured at 734 nm. ABTS antioxidant efficiency used gallic acid (GA) as the standard. The ABTS scavenging activity was expressed as the gallic acid equivalent antioxidant capacity (GAEAC).

For FRAP scavenging activity, the FRAP working solution (10 mM TPTZ, 20 mM FeCl_3_, and 300 mM sodium acetate) was prepared before the experiment. The 10 µl of natural peptide solution was mixed with 190 µl of the FRAP working solution. The reaction was incubated at room temperature in the dark for 25 min. The absorbance was measured at 593 nm. FRAP efficiency used FeSO_4_ as the standard. The FRAP scavenging activity was expressed as FeSO_4_ molar equivalent.

### Intracellular ROS determination

For intracellular ROS production, A549 cells were stimulated with a low dose of LPS that does not affect cell viability (Baek et al., 2020; Seo et al., 2015). The cells were pretreated with or without the IC_50_ dosage of natural peptides for 6 h, followed by 0.5 µg/ml LPS for 24 h. ROS produced within the intracellular compartment was evaluated by the cellular ROS detection kit (Abcam, Cambridge, United Kingdom) using fluorescence-based microplate platform. The working ROS staining solution was prepared by 4 μl of stock staining solution was freshly diluted with 2 ml of an assay buffer. The intracellular ROS was detected by adding 100 μl of working ROS staining solution into the A549 cells and incubating at 37 °C, 5% CO_2_ for 1 h. The fluorescence intensities for DCFH-DA were evaluated using λ_excited_ at 520 nm and measured λ_emitted_ at 605 nm using the microplate reader (*n* = 3). The intracellular ROS was expressed as relative ROS abundant intensity normalized to the fluorescence of the negative control or untreated cells.

### Sample preparation for proteomic analysis and LC-MS/MS setting

The effect of the natural peptides from the flower of *T. stans* on A549 cells was examined using a proteomic approach. The treated cells were extracted using 0.2% SDS, 20 mM DTT, 10 mM HEPES-KOH, pH 8.0 with protease inhibitor cocktail, followed by protein cleaned-up by acetone precipitation (1:5) at −20 °C for 16 h. The protein pellet was resolubilized by 0.15% RapidGest SF in 20 mM ammonium bicarbonate. The amount of 2 µg protein was reduced (5 mM of DTT at 72 °C for 30 min) and alkylated (25 mM IAA at RT in the dark for 30 min) prior to the digestion using trypsin at 37 °C for 6 h. The tryptic peptides were dried and reconstituted in 0.1% formic acid before being subjected to the LC-MS/MS.

Data acquisition in LC-MS runs was operated in the data-dependent acquisition mode. The protein expression was quantified by Progenesis^®^ QI for proteomics as described previously (Krobthong et al., 2021). The raw MS files were identified against the Uniprot human database (*Homo sapiens* retrieved 29 June 2021). The parameters were set to; MS tolerance, 20 ppm; MS/MS tolerance, 60 ppm; minimum fragment ion matches/peptide, 3; minimum fragment ion matches/protein, 3; minimum peptide ions/protein, 1; digest enzyme, trypsin; cysteine carbamidomethylation as fixed modification and methionine oxidation, asparagine and glutamine deamination as variable modifications.

### Bioinformatics analysis

The expression data were normalized and visualized using bioinformatics software. The normalization of relative protein expression data was analyzed using an R package, NormalyzerDE 1.1.13 (Willforss, Chawade & Levander, 2019), in which Quantile-normalization was applied to the relative expression data analysis, after adding “1” to all expression values to avoid errors upon log-transformation. The expression heatmap was obtained using MultiExperiment Viewer (MeV) for self-organizing tree clustering based on the expression data. Volcano plot analysis was obtained using plotting between the magnitude changes of proteins (log_2_ fold change) and statistical significance from ANOVA-test (-log ANOVA) in the experimental groups. Proteins having relative abundance with ANOVA < 0.05 between 6 LC-runs (*n* = 3) are considered differentially expressed.

### Statistical analysis

All experiments were carried out with three independent replicates (*n* = 3), and the results were reported as mean ± standard deviation (SD). One-way ANOVA with Tukey’s post-hoc test was performed using SPSS 18.0 software (SPSS Inc, Chicago, IL, USA) to analyze the statistical significance of differences between experimental groups for cell migration and invasion assays while paired Student’s *t*-test was used for intracellular ROS analysis, where asterisk (*, **, ***) indicated *p*-value of <0.05, <0.01 and <0.001, respectively. One-way analysis of variance was performed by Progenesis^®^ QI for proteomic analysis.

## Results

### *T. stans*-derived native peptide isolation and purification

**Figure 1:**
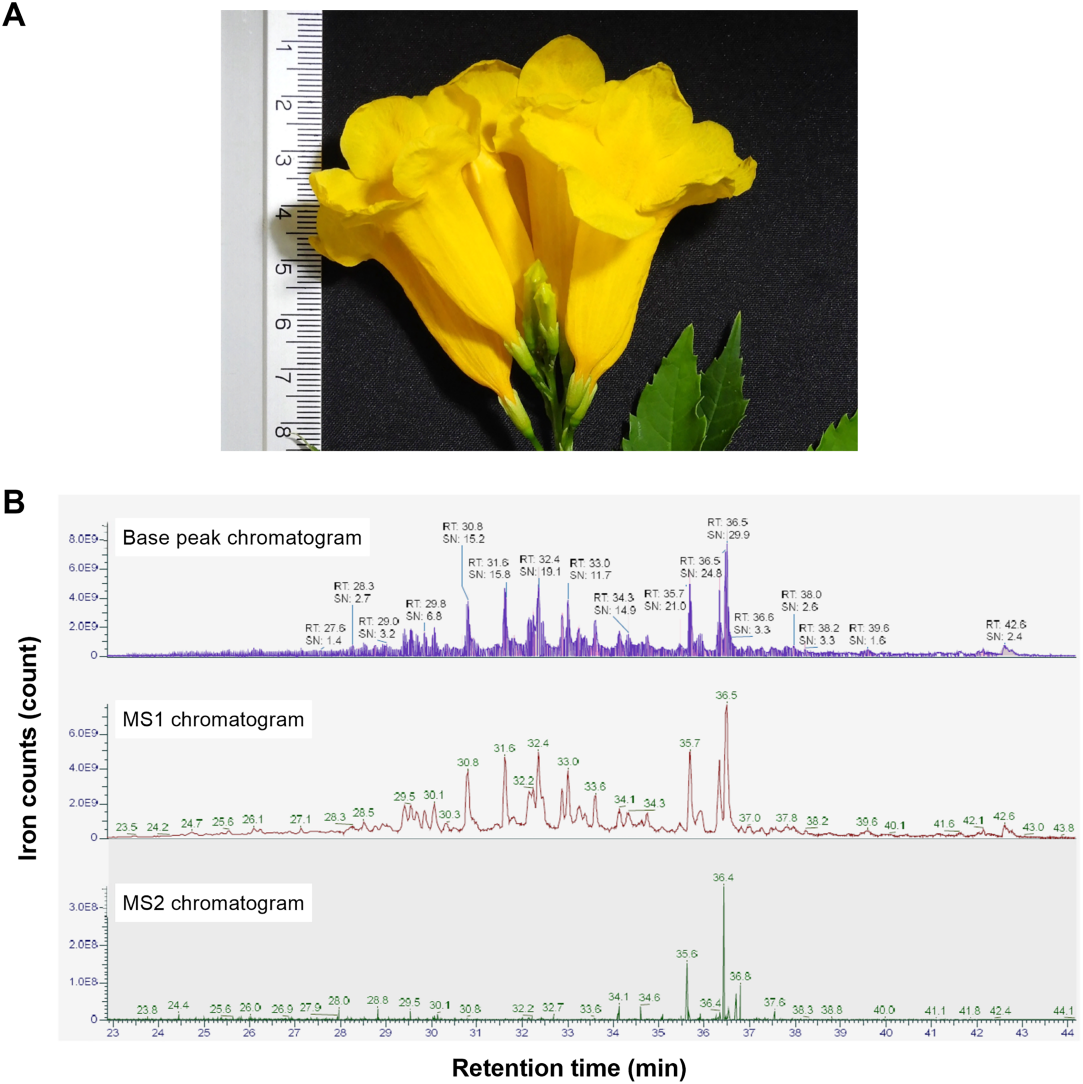
Stacked LC-MS/MS chromatograms of the natural peptides from *T. stans*. (A) The flower of *T. stans* before extraction by PHWE method for obtaining natural peptides. (B) The chromatogram of natural peptides analyzed by LC-MS/MS including TIC (total ion count), MS1 spectrum, and MS2 spectrum. Each chromatogram has been shown in ions count *vs* retention time.

In our study, we initially used the hot water extraction process to release the natural peptides in the flowers of *T. stans* (Fig. 1A). The natural peptides extracted from the flowers were purified using size-exclusion and reverse-phase approaches. After the purification steps, the eluent fractions were subjected to LC-MS/MS to explore the peptide profile (Fig. 1B). The natural peptides profile analysis revealed 126 peptide ions peaks. The highest peptide ion was eluted at 36.5 min with an intensity = 9.18e^9^ AU. Analysis of natural peptides, the value of peptide ions found in MS1 were verified using ion peaks of MS2 that resulted in peptides sequences with an ion-fragmentation evidence.

The *de novo* algorithm was used to predict the sequence originating from the peptide ion peak. The LC-MS/MS results contained 4,230 MS/MS spectra of +2 and +3 charged ions. Matching of the peptide spectrum revealed 126 peptides with lengths in the range of 6–44 amino acids and a molecular weight of 818.49–4,451.30 Da. The numbers reported in Fig. 1 correspond to the compounds shown in Table S1. The QC was done using qualitative analysis for different sampling batches. The peptide profile alignment of 3-batches revealed a high level of similarity (>95%).

### Cytotoxicity of natural peptides from *T. stans* in human cancer cell lines

**Figure 2:**
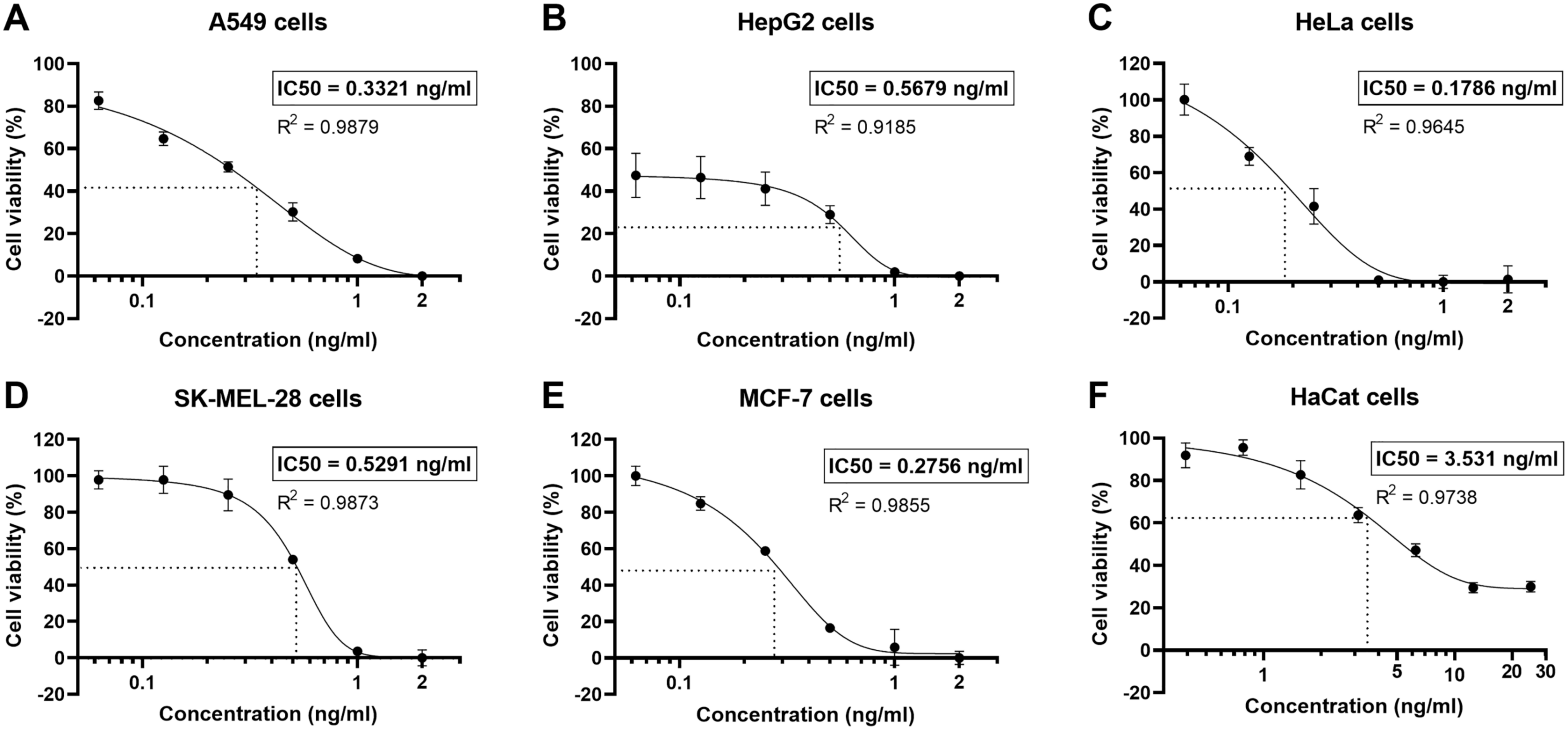
Natural peptides from *T. stans* showed selective cytotoxicity for human cancer cells. The human cancer (A) A549, (B) HepG2, (C) HeLa, (D) SK-MEL-28, and (E) MCF-7 cells were treated with 0.0625, 0.125, 0.25, 0.5, 1.0 and 2.0 ng/ml of natural peptides, while the immortalized human keratinocyte (F) HaCat cells were treated with 0.391, 0.781, 1.563, 3.125, 6.25, 12.5 and 25.0 ng/ml of natural peptides for 24 h before monitoring cell viability using MTT assay. The results were obtained from three independent experiments, each in triplicate.

The human non-small cell lung cancer, A549 cells, were undergone a cytotoxicity test. Cell viability was determined by MTT assay, using logarithmically growing A549 cells treated with various concentrations of the natural peptides for 24 h. The natural peptides induced cell cytotoxicity in the A549 cells (cell viability <60%) for concentrations >0.125 ng/ml (Fig. 2A). However, at the lowest tested concentration of 0.0625 ng/ml, the cell viability was 82.6 ± 3.4%. The percentage of A549 cell viability as the action of natural peptide concentrations was plotted in a sigmoidal curve. The sigmoidal curve of percent cell viability *vs* concentration showed the IC_50_ of 0.3321 ng/ml in A549 cells. In addition, we also evaluated the cytotoxicity of natural peptides from *T. stans* in the other human cancer (HepG2, HeLa, SK-MEL-28, and MCF-7) and immortalized keratinocyte (HaCat) cell lines (Figs. 2B–2F). The IC_50_ of natural peptides from *T. stans* in HepG2, HeLa, SK-MEL-28, MCF-7, and HaCat cells was 0.5679, 0.1786, 0.5291, 0.2756, and 3.531 ng/ml, respectively. The higher IC_50_ dosage in the HaCat cells, transformed from normal keratinocytes, indicated that natural peptides from *T. stans* have selective dose-dependent cytotoxicity for cancers. The IC_50_ dosage of A549 cells was chosen for intracellular ROS estimation and proteomics.

### Effect of natural peptides from *T. stans* on A549 cell migration and invasion

**Figure 3:**
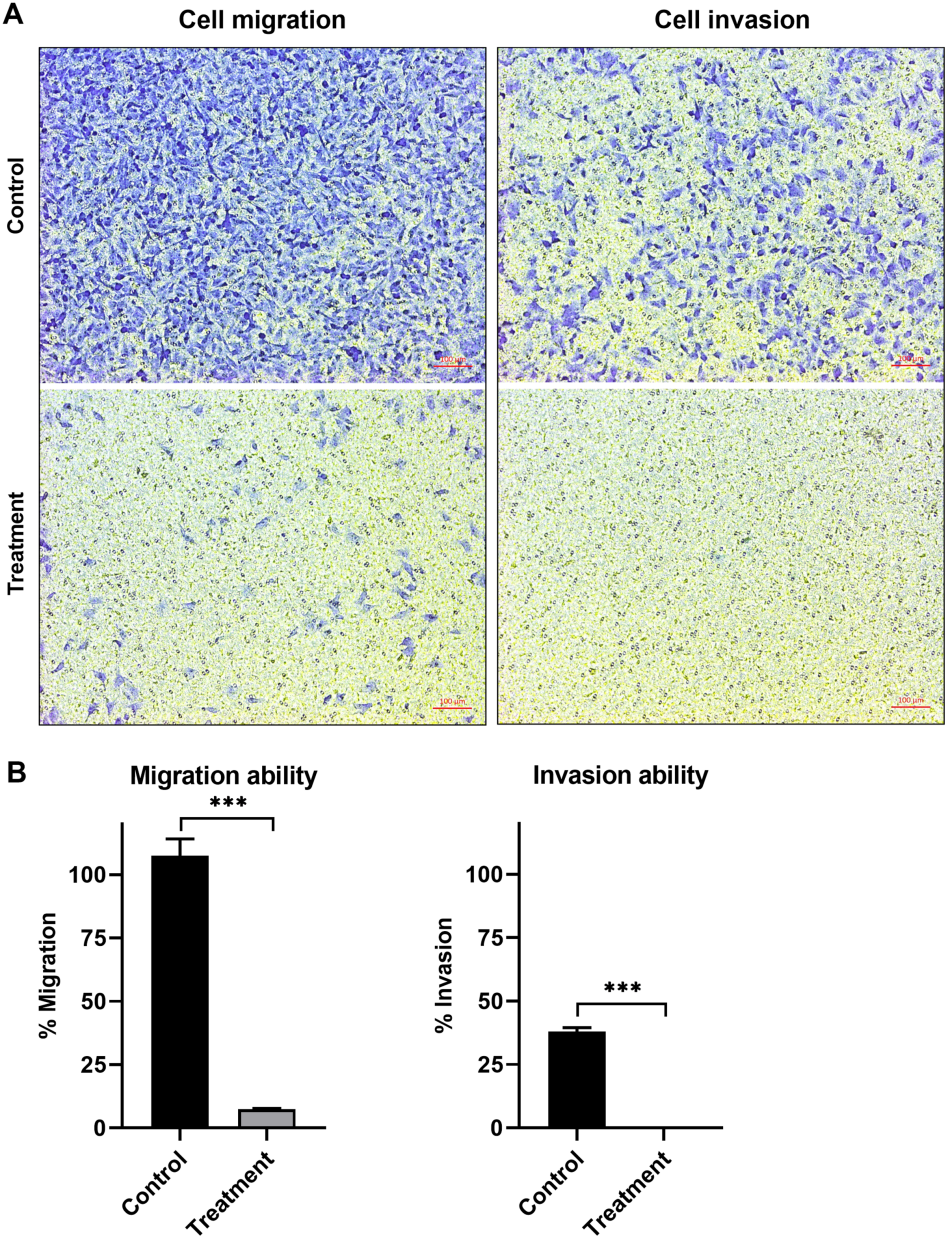
Natural peptides from *T. stans* markedly inhibited cell migration and invasion. (A) Migration (left panels) and invasion (right panels) assays of A549 cells treated with or without 0.0625 ng/ml of natural peptides for 24 h. The migrated and invaded cells were stained with crystal violet. Scale bar, 100 µm. (B) The percentage of migration and invasion of A549 cells treated with natural peptides. The cells were counted from five random fields for each condition in three independent experiments. The percentage of migrated cells treated with natural peptides was relative to the untreated control. The percentage of invaded cells treated with or without natural peptides was relative to that of the migratory control cells that were allowed to migrate across the uncoated-Matrigel membrane. The statistical analysis was conducted using one-way ANOVA. Asterisks (***) indicate *p*-value of <0.001.

The anti-migration ability of natural peptides from *T. stans* in A549 cells was determined by using a transwell chamber assay. Treatment with natural peptides at the lowest concentration of 0.0625 ng/ml markedly suppressed A549 cell migration (Fig. 3A, left panels) and showed a migration rate of less than 10% (*p* < 0.0001) (Fig. 3B, left panel). The Matrigel-coated transwell chamber assay was also performed to investigate the anti-invasion ability of natural peptides from *T. stans* in A549 cells. The result showed that natural peptides at 0.0625 ng/ml completely inhibited A549 cell invasion (Fig. 3A, right panels). The percentage of invaded cells of untreated condition was 38% ± 0.92, while A549 cells treated with natural peptides had completely lost their ability to invade through Matrigel-coated membrane (*p* < 0.0001) (Fig. 3B, right panel). Collectively, these results indicated that *T. stans* possesses natural peptides acting with both anti-migration and anti-invasion abilities in A549 cells.

### *In vitro* antioxidative activity using DPPH, ABTS and FRAP assays

**Figure 4:**
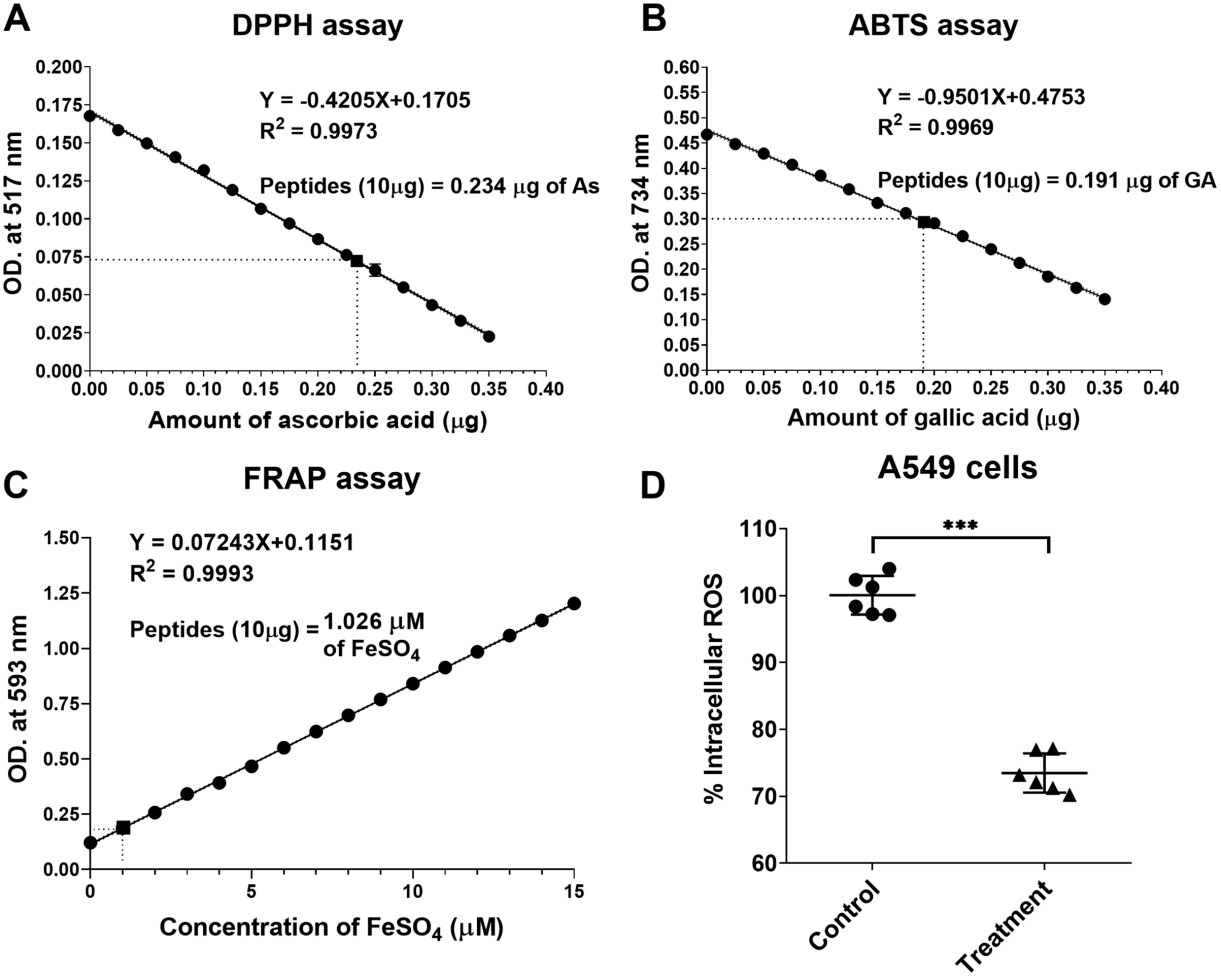
Natural peptides from *T. stans* exhibited antioxidative activity. (A) DPPH, (B) ABTS and (C) FRAP assays were performed to evaluate the free radical scavenging capacity of natural peptides in comparison with ascorbic acid, gallic acid, and FeSO_4_, respectively. (D) Intracellular ROS assay in the LPS-induced A549 cells. A549 cells were pretreated with or without 0.3321 ng/ml of natural peptides for 6 h, followed by 0.5 µg/ml LPS for 24 h before analyzing intracellular ROS. The data is represented as a mean ± SD from three independent experiments. The statistical analysis was conducted using paired Student’s *t*-test. Asterisks (***) indicate *p*-value <0.001).

The natural peptides from the flower of *T. stans* exhibited antioxidative ability. Ten micrograms of natural peptides were used to determine the free radical scavenging capacity relative to ascorbic acid, gallic acid, and FeSO_4_. The relative antioxidative abilities were determined *in vitro* using DPPH, ABTS and FRAP assays. The scavenging activity of 10 µg natural peptides from *T. stans* was 0.234 ± 0.02 µg of AsEAC for DPPH assay (Fig. 4A), 0.191 ± 0.03 µg of GAEAC for ABTS assay (Fig. 4B), and 1.026 µM of FeSO_4_ equivalent for FRAP assays (Fig. 4C), respectively. This result indicated that the natural peptides from the flower of *T. stans* are the intensive antioxidative peptides.

### Natural peptides from *T. stans* reduced intracellular ROS in LPS-induced A549 cells

We next investigated the antioxidative activity of natural peptides from the flower of *T. stans* in LPS-induced A549 cells. The relative ROS within the cells was measured by analyzing intracellular ROS with DCFH-DA. The qualitative analysis of relative ROS generation induced by LPS in A549 cells was presented in Fig. 4D. Compared to the control group, the intracellular ROS of the cells pretreated with natural peptides from *T. stans* at IC_50_ dosage significantly decreased by about 26.53 ± 2.91% (*p* < 0.0001) after treatment with LPS. The protective intracellular ROS production indicated that the natural peptides from the flower of *T. stans* are capable of reducing free radicals in the cells.

### Proteomic analysis

**Figure 5:**
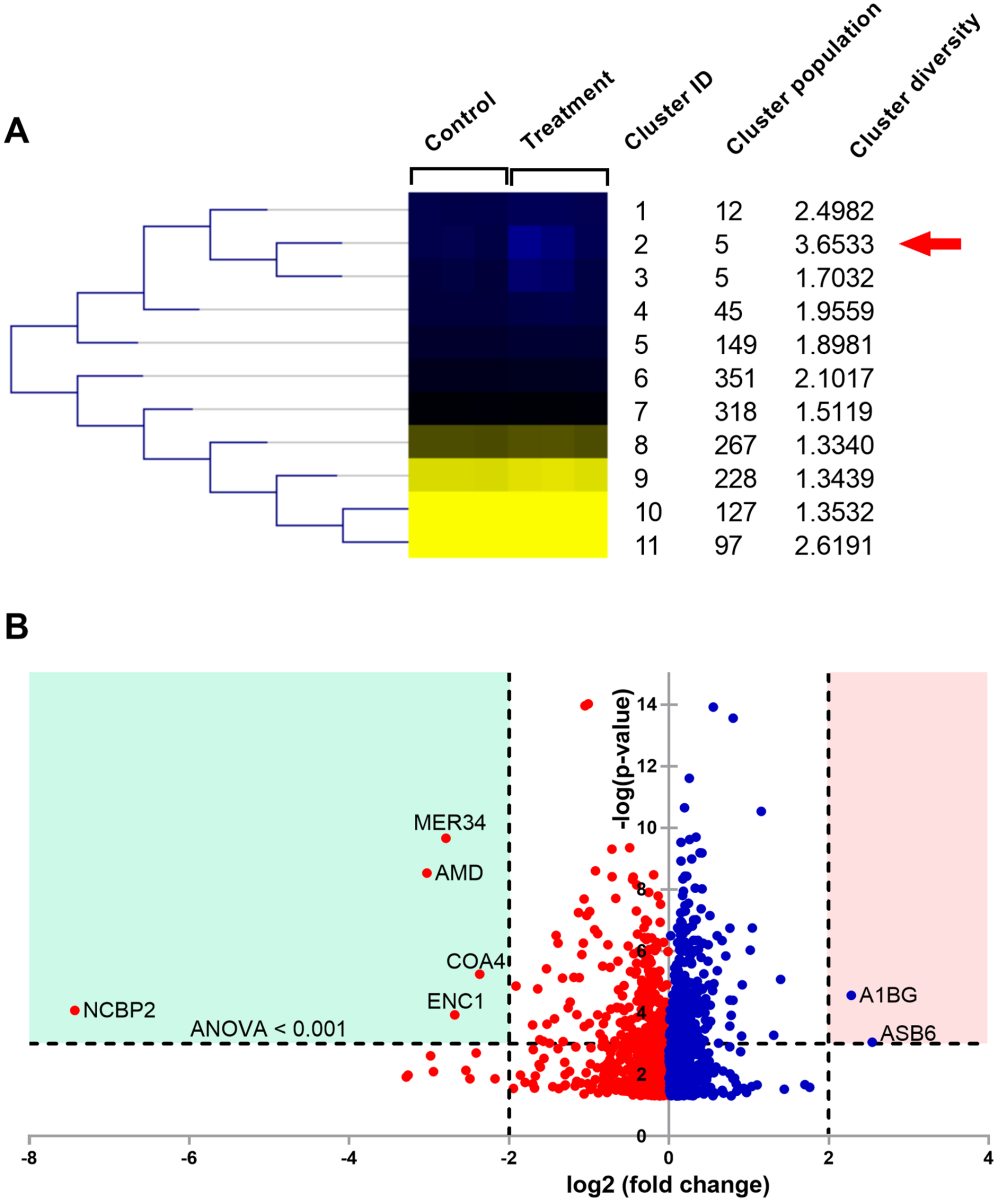
Proteomic analysis of A549 cells treated with natural peptides from *T. stans*. (A) SOTA dendrogram of protein abundance by sample. The heat-map shows the similarity of protein abundance in control and treatment groups. The differentially expressed proteins were grouped into 11 clusters with different protein expression profiles using centroid distance. (B) Volcano plot showing differential protein expressions. The points indicate different proteins that display both large magnitude fold changes (log_2_ fold change, *x*-axis) and high statistical significance (-log10 of ANOVA, *y*-axis). The dashed horizontal line shows the cutoff values for ANOVA <0.001. The two vertical dashed lines indicate the fold change >2 in down-regulated (blue region) and up-regulated (pink region) proteins.

Changes in the cellular proteins affected by the natural peptides from the flower of *T. stans* were studied using label-free quantitative proteomics. A total of 1,604 differentially expressed proteins were successfully identified. The relative expression ratios from both *T. stans* and control groups were analyzed using the Self Organizing Tree Algorithm (SOTA) to get a holistic view of the proteome expression pattern. After clustering, eleven distinct clusters were constructed based on the expression pattern (Fig. 5A).

The columns in the SOTA dendrogram represent the relative protein expression of the treatment (*n* = 3) and control (*n* = 3) groups. Meanwhile, the rows represent the average abundance level of the proteins in each cluster. Green denotes higher expression levels, while red denotes lower expression levels. The analysis revealed that cluster ID two has the highest cluster diversity (3.6533). There were five proteins in this cluster, including acetolactate synthase-like protein (ILVBL_HUMAN), calcium load-activated calcium channel (TMCO1_HUMAN), diacylglycerol kinase-α (DGKA_HUMAN), ectoderm-neural cortex protein 1 (ENC1_HUMAN), and nuclear cap-binding protein subunit 2 (NCBP2_HUMAN).

The differential expression ratio from the quantification of 1,604 proteins ranged from 0.01- to 7.43-fold changes as shown in Data S1. A total of 15 proteins exhibited a two-fold change between the two sample groups, while only seven proteins passed the two-fold change with ANOVA < 0.001 cutoffs. These results are illustrated as a volcano plot (Fig. 5B). Among these seven proteins, two were up-regulated while five were down-regulated. Identities of protein details that showed the differential expression of more than two-fold changes between *T. stans* and control are listed in Table 1.

**Table 1:** Identities and relative expression proteins of A549 cells treated with natural peptides from *T. stans*.

Accession (HUMAN)	Protein name	log_2_ intensity (AU)		log_2_ fold change	Anova *p*-value (10^−4^)
		Control ( $\bar{x}$)	Treatment ( $\bar{x}$)		
NCBP2	Nuclear cap-binding protein subunit 2	17.1869	10.2690	−7.4300	0.83219
AMD	Peptidyl-glycine alpha-amidating monooxygenase	21.3378	17.2993	−3.0269	0.00029
MER34	Endogenous retrovirus group MER34 member 1 Env polyprotein	20.0223	16.5028	−2.7889	0.00002
ENC1	Ectoderm-neural cortex protein 1	17.6641	14.6700	−2.6794	1.16420
COA4	Cytochrome c oxidase assembly factor 4 homolog, mitochondrial	19.5782	16.6167	−2.3661	0.05565
A1BG	Alpha-1B-glycoprotein	17.5864	20.6002	2.2819	0.26909
ASB6	Ankyrin repeat and SOCS box protein 6	15.0540	17.9550	2.5423	8.98046

According to Table 1 and Fig. 5B, the relative protein expressions of nuclear cap-binding protein subunit 2 (NCBP2_HUMAN), peptidyl-glycine alpha-amidating monooxygenase (AMD_HUMAN), endogenous retrovirus group MER34 member 1 Env polyprotein (MER34_HUMAN), ectoderm-neural cortex protein 1 (ENC1_HUMAN), and cytochrome *c* oxidase assembly factor 4 homolog (COA4_HUMAN) under the treatment condition were lower than that of the control by 7.430, 3.027, 2.789, 2.679 and 2.366 (log_2_) fold changes, respectively. Meanwhile, the relative protein expressions of ankyrin repeat and SOCS box protein 6 (ASB6_HUMAN) and alpha-1B-glycoprotein (A1BG_HUMAN) in the treatment group were higher than that of the control by 2.542 and 2.282 (log_2_) fold changes, respectively.

## Discussion

The biological compounds derived from *T. stans* are essential in discovering novel therapeutic bioactive compounds and synthesizing new potential drugs. Biological components such as glycosides, tannins, flavonoids, alkaloids, saponins, and coumarins from medicinal plants can synergize and produce desirable pharmacological effects for health (Anand & Basavaraju, 2021; Milugo et al., 2013). However, the natural peptides from medicinal plants, especially *T. stans*, are studied less intensively. Pressurized hot water extraction (PHWE) should be performed to release the natural peptides from *T. stans* entirely. The easiest and most robust approach to obtaining natural peptides is column-based ultrafiltration, a membrane-based filtration used in size-fractionation. In this study, we used ultrafiltration with a nominal 3-kDa cut-off to remove the intact proteins and large molecular weight compounds and obtain only the natural peptides, consistent with the previous report which showed that a <3-kDa molecular weight cut-off provided great scavenging radicals abilities (Krobthong & Yingchutrakul, 2020). A combination with the SPE technique also improved the efficiency of removing metabolites and concentrating the natural peptides. Natural peptide extraction was confirmed using the *de novo* algorithm by LC-MS/MS. We used the MS2 spectra to distinguish peptides and metabolites. The QC of natural peptides extraction was done using LC-MS/MS. This approach used qualitative and quantitative multiplex analysis in different batches to monitor individual variations caused by production techniques and starting material (Dallas et al., 2014; Nielsen et al., 2017).

The anti-proliferative activity of methanolic extract from the flowers of *T. stans* has been reported in Vero and Hep-2, the human larynx carcinoma cell lines. The methanolic extract reduced 50% the growth of the cells at a concentration of 250 µg/ml (Kameshwaran et al., 2012). The cytotoxicity of methanolic extract from the leaves and flowers of *T. stans* was also studied in A549 cells. A concentration range of 20–100 µg/ml showed toxicity to the cells (cell viability <50%) (Robinson et al., 2017). However, our results revealed superior cytotoxicity to A549 cells when using PHWE for natural peptides extraction, having a more than 1,000-fold increase in the cytotoxic activity (Fig. 2A). The higher cytotoxicity of natural peptides extracted by the PHWE-based method is likely attributed to the capacity of peptides to form alpha-helix structure and insert into the cell membrane, pore-forming and inducing membrane disruption (Ma, Mahadevappa & Kwok, 2017). In contrast, the extraction with organic solvents alone, such as methanol or ethanol, obtains other bioactive compounds that cannot execute pore formation, decreasing cytotoxic activity. This result suggests that natural peptides have more potential against the A549 cells. Moreover, we also evaluated the cytotoxicity of natural peptides against the other human cancer (HepG2, HeLa, SK-MEL28, and MCF-7) and immortalized (HaCat) cells to visualize global cytotoxicity (Figs. 2B–2F). The natural peptides from *T. stans* showed the suppression of cell growth in all cancer cell types at nanogram ranges, consistent with the previous report which showed that natural peptides isolated from the sponge *Homophymia* sp. are toxic to several cancer cells with the IC_50_ values ranging from 2 to 100 nM (Zampella et al., 2009). This result indicates that natural peptides from *T. stans* have a broadly anti-proliferative activity in human cancer cell types. It is noted that all cancer cell lines had the IC_50_ of the natural peptides from *T. stans* lower than the immortalized cells. The IC_100_ of natural peptides (1 ng/ml) in all cancer cell lines was not toxic to the HaCat cells as transformed from normal keratinocytes (Cell viability >90%), indicating that natural peptides from *T. stans* are selectively cytotoxic to cancer cells.

In addition to an anti-cancer property, we further investigated the anti-metastatic ability of natural peptides from *T. stans* in A549 cells. Metastasis is the characteristic of cancer cells to spread to other parts of the body. Cancer cells can migrate and invade through the extracellular matrix and establish secondary tumors. Thereby they lead to cancer-related death rather than primary tumor (Gupta & Massagué, 2006). Lung cancer is the leading cause of cancer death with a poor prognosis (Berghmans, Paesmans & Sculier, 2011; Popper, 2016). Most lung cancer mortality is a result of metastasis. In this study, a transwell chamber assay was used to investigate the anti-migration and anti-invasion effects of the natural peptides from *T. stans* on A549 cells. Treatment with natural peptides at the lowest concentration of 0.0625 ng/ml showed effective inhibition of cell migration and invasion in A549 cells (Fig. 3). Loss of the competency of cell motility indicates that natural peptides from *T. stans* exhibit anti-migration and anti-invasion activities. Furthermore, to investigate whether the highest peptide eluted at 36.5 min (Fig. 1B) possesses the inhibitory effects on cell growth, migration and invasion, A549 cells were treated with the synthetic peptide, relating to the highest peak. It is noted that the synthetic peptide could not reduce cell growth, migration and invasion of A549 cells (Fig. S1). The ineffectiveness of a single synthetic peptide suggests that the highest peak peptide is not the bioactive peptide or may require the synergy of other natural peptides.

Although several bioactive compounds from *T. stans* have shown antioxidative ability (Aguilar-Santamaría et al., 2009; Alonso-Castro et al., 2010; Anand & Basavaraju, 2021; Marzouk et al., 2006; Salem et al., 2013), the free radical scavenging capacity of natural peptides has not been reported. In the *in vitro* assays, we underlined that the natural peptides from *T. stans* could be as efficient as other known antioxidants such as ascorbic acid, gallic acid, and FeSO_4_ (Figs. 4A–4C) (Abramovj et al., 2018). Moreover, to evaluate the antioxidative activity of the natural peptides from *T. stans* on A549 cells, a low dose of LPS (0.5 µg/ml) was used to stimulate intracellular ROS production. It is noted that the cell viability of A549 cells pretreated with the IC_50_ dosage of natural peptides was no significant change after stimulation with or without LPS (Fig. S2), indicating the only LPS-mediated ROS activation. This result is consistent with previous reports, which showed that the low LPS induces ROS production without cell toxicity (Baek et al., 2020; Seo et al., 2015), but the high LPS greater than 5 µg/ml is toxic to the cells (Gonçalves et al., 2016; Sangaran et al., 2021). We also showed that treatment with natural peptides was able to reduce intracellular ROS in the LPS-induced A549 cells (Fig. 4D), suggesting antioxidative ability within intracellular compartments.

The use of proteomics in exploring molecular pathways is a broad and useful technique for the initial understanding of cell response and eventual identification of new disease targets for therapeutic interventions. Herein, proteomics was applied to study cell motility inhibition and oxidative stress prevention from natural peptides derived from *T. stans*. This study is the first report on analyzing proteome changes when A549 cells were exposed to the natural peptides from *T. stans*. The volcano plot revealed that five proteins (NCBP2, AMD, MER34, ENC1, and COA4) were down-regulated, but two proteins (A1BG and ASB6) were up-regulated under the treatment condition (Fig. 5B). In addition, it is noted that the mRNA levels of NCBP2 and ASB6 in the A549 cells treated with natural peptides were also correlated with proteomic analysis (Fig. S2). Regarding the down-regulated proteins, NCBP2 is the lowest suppressed protein in the *T. stans* treatment group. NCBP2, the 20 kDa subunit of the nuclear cap-binding complex, directly binds to the 5′-end of RNA cap, and it is stabilized by NCBP1, an 80 kDa subunit protein, to orchestrate post-transcriptional processes (Dou et al., 2020; Gebhardt et al., 2015). They are the high conservative proteins from plants to humans. NCBP1 and NCBP2 regulate biological properties by participating in transcription, splicing, transcript export, translation, histone modification, RNA modification, and spliceosome assembly. Overexpression of NCBP1 and NCBP2 are associated with lung cancer progression. Bioinformatics analysis of The Cancer Genome Atlas (TCGA) dataset showed that NCBP1 (Zhang et al., 2019) and NCBP2 (Xie et al., 2018) are highly expressed in lung cancer tissues and their expression levels are associated with poor prognosis. NCBP1, a partner of NCBP2, promotes proliferation, migration, and wound healing of lung cancer cell lines. Furthermore, suppression of NCBP1 expression markedly reduces cell growth and motility of lung cancer cells (Zhang et al., 2019).

The other suppressed proteins have been shown to support tumorigenesis in several cancers. AMD, an enzyme that catalyzes the conversion of terminal-glycine peptides to α-amidated peptides (Eipper et al., 1993), has been reported to be overexpressed in lung cancer cell lines, and AMD inhibitors can suppress the growth of cancer cells (Iwai et al., 1999; Vos et al., 1996). A recent study also showed that the AMD expression is up-regulated under hypoxic conditions and necessary for angiogenesis in glioblastoma (Soni et al., 2020). Moreover, suppression of AMD expression significantly decreases tumor size and increases survival in the xenograft mice. Although MER34, a protein encoded by the retroviral envelope (Env) gene which integrates into the genome of a human ancestor (de Parseval & Heidmann, 2005), is highly expressed in normal placenta and embryonic stem cells (Mi et al., 2000), bioinformatics and immunohistochemistry analyses demonstrated that both mRNA and protein levels of MER34 are highly expressed in ovarian cancer (Heidmann et al., 2017), respectively.

ENC1, an actin-binding protein that is essential for gastrulation and nerve formation (Hernandez et al., 1997), has been reported to be overexpressed in the colon (Cui et al., 2021), breast (Zhou et al., 2020), and lung cancers (Wu et al., 2021). A cohort study of TCGA dataset showed that overexpression of ENC1 is associated with tumor progression and poor prognosis. Suppression of ENC1 expression inhibits the growth, migration and invasion of colon, breast, and lung cancer cell lines (Cui et al., 2021; Wu et al., 2021; Zhou et al., 2020). Moreover, the knockdown of ENC1 significantly down-regulates the expression of mesenchymal markers (matrix metalloproteinase and N-cadherin) and up-regulates the expression of epithelial marker (E-cadherin) in lung cancer cell lines (Wu et al., 2021). Although the cancer-based study of COA4, a mediator protein in the assembly process of the cytochrome *c* oxidase complex (Watson & McStay, 2020), is lacking, overexpression of its related protein (COA1) has also been shown in colorectal cancer. The microarray assay showed that overexpression of COA1 in colorectal cancer tissues is associated with poor prognosis, and suppression of COA1 expression reduces the growth of colorectal cancer cell lines (Xue et al., 2020). Here, we showed that natural peptides from *T. stans* inhibited the growth, migration and invasion of A549 cells by suppressing the expression of cancer-promoting proteins (NCBP2, AMD, MER34, ENC1, and COA4).

On the other hand, A1BG and ASB6 were up-regulated in the *T. stans* treatment group (Fig. 5B). A1BG is a secretory glycoprotein in the bloodstream (Udby et al., 2004). Although A1BG has been reported to be overexpressed in plasma patients with type two diabetes mellitus (Galazis et al., 2013), cervix (Jeong et al., 2008), and lung cancers (Liu et al., 2012), its functional role is unknown and further needs to be investigated. As A1BG was up-regulated in the treatment group, it suggests that the natural peptide from *T. stans* may inhibit the secretion of A1BG into the culture media, resulting in overexpressed-A1BG in the cells. The highest overexpressed protein, ASB6, is an E3 ubiquitin-protein ligase targeting protein for proteasome degradation (Kohroki et al., 2005). The up-regulation of ASB6 is associated with the elevation of intracellular ROS in oral cancer cells (Hung et al., 2009). A recent study also showed that overexpression of ASB6 can decrease intracellular ROS and endoplasmic reticulum stress in the deprived media-induced stress of oral cancer cells (Hung et al., 2019). Therefore, the reduction of intracellular ROS in the LPS-stimulated cells (Fig. 4D) is likely attributed to the up-regulation of ASB6, which is involved in the damaged-protein degradation process, reducing oxidative stress within the cells.

## Conclusions

We showed the series methods of PHWE, size exclusion chromatography, and SPE to extract and purify the natural peptides from the flower of *T. stans*. The peptide ion peaks and their sequences were identified by LC-MS/MS. The natural peptides exhibited anti-proliferative, anti-migration and anti-invasion activities on A549 cells. They also reduced intracellular ROS in the LPS-induced A549 cells. Proteomics revealed the seven proteins affected by the natural peptides. The cancer-promoting proteins (NCBP2, AMD, MER34, ENC1, and COA4) were suppressed, while the secretory glycoprotein (A1BG) and ROS-reducing protein (ASB6) were overexpressed in the treatment group. It is noted that the anti-proliferative and anti-metastatic activities of natural peptides may be attributed to the suppression of several cancer-promoting proteins, but their antioxidative activity may result from an up-regulation of ROS-reducing protein. Our finding suggests that natural peptides from *T. stans* are viable for being the new potential anti-cancer and antioxidative agents.

## Supplemental Information

10.7717/peerj.13693/supp-1 Supplemental Information 1 Chromatographic analysis of identified peptide ion peaks from *T. stans*. A total of 126 peptide ions with peak area measurement, sequence, mass deviation, and molecular weight were showed.

10.7717/peerj.13693/supp-2 Supplemental Information 2 Synthetic peptide from the highest peak (RT of 36.5 min) could not reduce cell viability, migration, and invasion of A549 cells. (A) Cell viability of A549 cells treated with various concentrations of the synthetic peptide from the highest peak (RT of 36.5 min). (B) Cell migration of A549 cells treated with 25 µg/ml synthetic peptide.

10.7717/peerj.13693/supp-3 Supplemental Information 3 LPS did not change cell viability, and the relative gene expressions of NCBP2 and ASB6 were correlated with proteomics. (A) Cell viability of A549 cells pretreated with 0.3321 ng/ml of natural peptides for 6 h, stimulated with or without 0.5 µg/ml LPS for 24 h. (B) Relative gene expressions of NCBP2 and ASB6 in A549 cells pretreated with or without 0.3321 ng/ml of natural peptides for 6 h, followed by 0.5 µg/ml LPS for 24 h. The expression of NCBP2 and ASB6 was normalized with that of 18S rRNA and shown as relative expression. The expression of NCBP2 and ASB6 in treated cells was relative to that obtained from the untreated control cells, which was arbitrarily set as 1. * and *** for *p*-values <0.05 and <0.001, respectively. (B) The melt curve analysis of 18S rRNA, NCBP2, and ASB6.

10.7717/peerj.13693/supp-4 Supplemental Information 4 The differential protein expression profiles of A549 cells treated with natural peptides from *Tecoma stans*.

10.7717/peerj.13693/supp-5 Supplemental Information 5 Raw data of cell viability.

10.7717/peerj.13693/supp-6 Supplemental Information 6 Raw data of cell migration and invasion.

10.7717/peerj.13693/supp-7 Supplemental Information 7 Raw data of migration assay.

10.7717/peerj.13693/supp-8 Supplemental Information 8 Raw data of Invasion assay.

10.7717/peerj.13693/supp-9 Supplemental Information 9 Raw data of DPPH, ABTS, FRAP, and ROS.

10.7717/peerj.13693/supp-10 Supplemental Information 10 The viability, migration and invasion of A549 cells treated with synthetic peptide from RT of 36.5.

10.7717/peerj.13693/supp-11 Supplemental Information 11 The oligonucleotides used for qRT-PCR and the relative gene expressions of A549 cells treated with natural peptides from *Tecoma stans*.

## Additional Information and Declarations

### DNA Deposition

The following information was supplied regarding the deposition of DNA sequences: The protein sequences are available at GenBank (Table S1).

### Data Availability

The following information was supplied regarding data availability: The raw measurements are available in the Supplemental Files.
