# Supplementary material for: Evaluation of potential anti-metastatic and antioxidative abilities of natural peptides derived from Tecoma stans (L.) Juss. ex Kunth in A549 cells
Source: PeerJ. 2022 Jul 6;10:e13693. doi: 10.7717/peerj.13693 (PMC9270879; doi:10.7717/peerj.13693)
Supplement: Supplemental Information 1 — A total of 126 peptide ions with peak area measurement, sequence, mass deviation, and molecular weight were showed. [file peerj-10-13693-s001.docx]

**Supplemental Table S1.** Chromatographic analysis of identified peptide ion peaks from *T. stans*. A total of 126 peptide ions with peak area measurement, sequence, mass deviation, and molecular weight were showed.

| **Peptide ions No.** | **Peak Area** | **Sequence** | **Mass deviation (ppm)** | **Molecular weight (Da)** |
| --- | --- | --- | --- | --- |
| 1 | 1.94E+10 | EGNELLR | 4.8 | 829.4294 |
| 2 | 2.26E+10 | TSVDLEETGR | 4.0 | 1105.5251 |
| 3 | 1.01E+10 | LATTGAFNAR | 4.1 | 1020.5352 |
| 4 | 1.93E+10 | PNAENFDPTAR | 4.8 | 1230.563 |
| 5 | 1.44E+10 | PNAENFDPTAR | 4.8 | 1230.563 |
| 6 | 5.77E+09 | DGSAEFSSLLEER | 6.9 | 1438.6575 |
| 7 | 8.09E+09 | LYENLSNLDHAAK | 4.1 | 1486.7415 |
| 8 | 7.20E+09 | VATVSLPR | 5.0 | 841.5021 |
| 9 | 7.24E+09 | TNSVLQELR | 3.3 | 1058.572 |
| 10 | 5.05E+09 | LHGYVLDDPANQELER | 5.6 | 1867.9065 |
| 11 | 4.99E+09 | TACLDLTVK | 5.4 | 962.5107 |
| 12 | 4.19E+09 | SSDVTTPTLGK | 3.5 | 1104.5662 |
| 13 | 1.20E+10 | LTYYTPDYETK | 5.5 | 1392.6448 |
| 14 | 7.39E+09 | YEDNFDTTSDVAVFVTTTDKK | 0.1 | 2395.1067 |
| 15 | 4.05E+09 | TSVLDGLK | 5.0 | 831.4702 |
| 16 | 2.09E+09 | LGGLGTVRGVP | 4.5 | 1024.6029 |
| 17 | 2.33E+09 | LQFNESFAEFNQ | 7.9 | 1472.6572 |
| 18 | 2.63E+09 | PLLSRSNLP | 4.3 | 995.5764 |
| 19 | 2.19E+09 | LSGLLYEETR | 3.5 | 1179.6135 |
| 20 | 4.50E+09 | YEDNFDTTSNVVVTVTPTDKK | 5.2 | 2372.1382 |
| 21 | 4.40E+09 | HEEPVVLLGNTEAPLNPK | 10.0 | 1956.0315 |
| 22 | 3.72E+09 | TFSEYPLPGR | 5.1 | 1165.5767 |
| 23 | 2.63E+09 | VYGTGSLALYEK | 5.7 | 1299.6711 |
| 24 | 2.40E+09 | EFNGLGDCGLTK | 5.5 | 1252.5757 |
| 25 | 1.91E+09 | LSELEAALQR | 6.5 | 1128.6138 |
| 26 | 1.94E+09 | LNVELTVEER | 5.3 | 1200.635 |
| 27 | 2.45E+09 | GLAYDLSDDQQDLTR | 5.1 | 1708.7903 |
| 28 | 1.81E+09 | TGMVWPTLGK | 5.8 | 1088.5688 |
| 29 | 1.61E+09 | TAGGLLLTEATK | 5.5 | 1173.6604 |
| 30 | 1.72E+09 | LTGLALQGALEK | 4.5 | 1212.7078 |
| 31 | 1.98E+09 | EVDYLLR | 4.3 | 906.481 |
| 32 | 1.33E+09 | KGGSNDTLQHEATAAALR | 2.6 | 1838.9233 |
| 33 | 1.77E+09 | LLESPAPLGLSR | 5.5 | 1251.7188 |
| 34 | 2.28E+09 | DPLEANLALRR | 4.6 | 1266.7043 |
| 35 | 3.79E+09 | DSPSEGPDTTSNVAVMVTTTDKK | -0.2 | 2379.1111 |
| 36 | 2.09E+09 | QGLLNLEVK | 3.6 | 1012.5917 |
| 37 | 2.67E+09 | TLYPDGFVR | 4.1 | 1066.5447 |
| 38 | 1.94E+09 | LEELEEELEAER | 5.8 | 1487.699 |
| 39 | 1.79E+09 | LLGADTSVDLEEWK | 3.0 | 1574.7827 |
| 40 | 1.58E+09 | LPPPLPEYGGK | 6.7 | 1166.6335 |
| 41 | 1.87E+09 | NVLEVAKHLGESTVR | 10.0 | 1650.9053 |
| 42 | 1.27E+10 | LVLEVAQHLVSTWR | -3.0 | 1649.9253 |
| 43 | 3.31E+09 | LEDEQALALQLQK | 5.9 | 1497.8037 |
| 44 | 1.26E+09 | EDYGHQTDSSQVLK | -4.9 | 1605.7271 |
| 45 | 1.78E+09 | AVDSLVPLGR | 4.6 | 1025.5869 |
| 46 | 1.13E+09 | KDLDDLELTLAK | 4.9 | 1372.7449 |
| 47 | 1.29E+09 | LTAADDLELTLAK | 4.7 | 1372.7449 |
| 48 | 1.30E+09 | LTTLEVESSDTLDNVK | 6.3 | 1762.8835 |
| 49 | 2.45E+09 | LVLSLGQDVEAGK | 5.8 | 1327.7346 |
| 50 | 1.77E+09 | VVETFSEYPPLGR | 5.5 | 1492.7561 |
| 51 | 2.43E+09 | FLVELR | 3.4 | 775.4592 |
| 52 | 1.12E+09 | EAGTVEVAEVLYNR | 5.7 | 1548.7783 |
| 53 | 1.38E+09 | QGVLTLELR | 4.4 | 1027.6025 |
| 54 | 1.99E+09 | LDLSPFK | 2.8 | 818.4538 |
| 55 | 1.14E+09 | TYYSSLVLTR | 5.8 | 1201.6343 |
| 56 | 1.19E+09 | FEEKDGLDYAAVTVQLWGPR | -1.6 | 2293.1377 |
| 57 | 1.29E+09 | DFQLEAALSK | 4.2 | 1120.5764 |
| 58 | 8.78E+08 | AFVLAALDGR | 4.8 | 1031.5764 |
| 59 | 1.05E+09 | FTQAGSEVSALLGR | 5.4 | 1434.7466 |
| 60 | 9.62E+08 | HALLLYDDLSK | 4.6 | 1286.687 |
| 61 | 1.05E+09 | KNAEAEDVSLFK | -2.6 | 1349.6826 |
| 62 | 9.78E+08 | TDELLWAGLTDQHVK | 5.8 | 1724.8733 |
| 63 | 9.83E+08 | LLLPHVDVQLK | 4.8 | 1273.7759 |
| 64 | 2.55E+09 | PSELESLLGKP | 6.1 | 1168.6338 |
| 65 | 1.28E+09 | VLDSQPLKLPVGPETLGR | 5.0 | 1918.0886 |
| 66 | 1.00E+09 | LALNLSLR | 4.1 | 898.56 |
| 67 | 9.49E+08 | WAQFLADLGCTLPDTEK | 5.2 | 1906.9136 |
| 68 | 9.18E+08 | LTLSALVDGK | 3.5 | 1015.5913 |
| 69 | 1.12E+09 | QGLLGLNLAEK | 4.4 | 1154.6658 |
| 70 | 1.31E+09 | LYGCLYVALGQK | 5.2 | 1326.7004 |
| 71 | 9.27E+08 | VVDLLAPYAK | 3.9 | 1087.6277 |
| 72 | 1.34E+09 | LGLEEYLER | 4.3 | 1120.5764 |
| 73 | 8.97E+08 | LTNLLELEVK | 4.4 | 1170.686 |
| 74 | 9.38E+08 | ADLLAYLK | 4.8 | 905.5222 |
| 75 | 1.93E+09 | YYAATVSTLNDVLAK | 6.1 | 1627.8457 |
| 76 | 2.57E+09 | WLAYGELLNGR | 6.0 | 1290.6721 |
| 77 | 1.64E+09 | LDLNLDLSK | 4.2 | 1029.5706 |
| 78 | 1.03E+09 | FDLNTVLAAK | 4.6 | 1090.6023 |
| 79 | 8.65E+08 | FVQAGSEVSALLGR | 6.4 | 1432.7673 |
| 80 | 7.34E+08 | NPAVATANLLETATPVVDKG | 7.3 | 1980.0527 |
| 81 | 8.40E+08 | VPNNNAWAYATNFVPGK | 4.1 | 1861.9111 |
| 82 | 8.64E+08 | FDSLEQLDEFSR | 3.9 | 1484.6782 |
| 83 | 7.22E+08 | LGEYGFQNALLVR | 4.9 | 1478.7881 |
| 84 | 9.64E+08 | NTFPQDQLATLTGR | 9.6 | 1560.7896 |
| 85 | 8.11E+08 | LVEAQNALVAGSAVFQK | 4.6 | 1743.9519 |
| 86 | 8.30E+08 | LTVDFLQDK | 3.0 | 1077.5706 |
| 87 | 9.38E+08 | MVFKLSYGGPDLVMEQLGK | 9.3 | 2111.0793 |
| 88 | 6.67E+08 | FLFGCAEALYK | 4.8 | 1260.6213 |
| 89 | 5.79E+08 | VSLAVLNPYLK | 5.2 | 1215.7227 |
| 90 | 8.69E+08 | LYSLLFR | 3.5 | 910.5276 |
| 91 | 2.83E+09 | DGPRLSFLAYKPTDT | 6.9 | 1679.8518 |
| 92 | 3.48E+09 | NSYLEVLLK | 4.1 | 1077.6069 |
| 93 | 2.58E+09 | QADTAELFFEDVR | 6.1 | 1539.7205 |
| 94 | 1.20E+09 | QSLLQAELEELR | 5.7 | 1427.762 |
| 95 | 7.38E+08 | DFLAGGLAAAVSK | 4.9 | 1218.6609 |
| 96 | 9.08E+08 | LLWQFLK | 4.8 | 946.564 |
| 97 | 6.87E+08 | LTLSALLDGK | 3.9 | 1029.6069 |
| 98 | 7.43E+08 | LDLFALK | 3.5 | 818.4902 |
| 99 | 6.96E+08 | NLAVPNSVVDLVVQVSKK | 8.4 | 1908.1042 |
| 100 | 9.26E+08 | DPLEANLALR | 4.3 | 1110.6033 |
| 101 | 9.44E+08 | LFPLFDR | 4.0 | 906.4963 |
| 102 | 6.72E+08 | LDENVATLALEGLK | 5.3 | 1484.8086 |
| 103 | 6.63E+08 | LNLGWLEK | 4.2 | 971.544 |
| 104 | 6.44E+08 | GSYVYPLDLFN | -0.6 | 1286.6182 |
| 105 | 4.71E+08 | LALFNPDVSWDR | 5.0 | 1431.7146 |
| 106 | 5.82E+08 | LFGVTTLDLVR | 4.6 | 1232.7129 |
| 107 | 1.55E+09 | ELLDLVLDR | 4.4 | 1084.6128 |
| 108 | 9.68E+08 | SEDLLAAFR | 3.0 | 1020.524 |
| 109 | 1.12E+09 | VALTGLTVCPAAAPR | -5.7 | 1438.7966 |
| 110 | 7.99E+08 | LFLPDRPSLT | 4.5 | 1157.6445 |
| 111 | 6.98E+08 | VPFLFTLK | 3.8 | 963.5793 |
| 112 | 6.01E+08 | LGFNGFSLTLK | 6.0 | 1195.6602 |
| 113 | 6.10E+08 | DFPLTGYVELR | 4.5 | 1308.6714 |
| 114 | 5.73E+08 | LFNVLGEPLDNLRPDLSG | 5.5 | 1968.0315 |
| 115 | 5.70E+08 | QPQAPPNAPVAAVAAVGKAADAAPVVEEKTEFDVVELEVFALHG | -2.2 | 4451.3013 |
| 116 | 5.32E+08 | LTLDASLVAGFTVR | 4.4 | 1461.8191 |
| 117 | 8.62E+08 | WHTVLSTVGHLKLYYSK | 7.3 | 2031.0942 |
| 118 | 4.72E+08 | VPLLVTGNDFSTLYALPLR | 5.5 | 2088.1619 |
| 119 | 7.25E+08 | KNTLGFVDLLR | -3.3 | 1274.7346 |
| 120 | 5.03E+08 | ETFLKSYLVDLTEEQLGKEVDYLLR | 1.1 | 3000.5693 |
| 121 | 5.17E+08 | PTPLQEGLLVGLSSQAAAAALK | 4.3 | 2134.1997 |
| 122 | 5.48E+08 | MVLFKSYGRDLTEEQLGKEVDYLLR | 6.4 | 3001.5581 |
| 123 | 5.96E+08 | LTPPPLSYGGPDLTEEQLGKEVDYLLR | 9.4 | 2999.5491 |
| 124 | 2.70E+09 | VDPPPLSYLVDLTEEQLGKEVDYLLR | 1.3 | 3000.5693 |
| 125 | 1.49E+09 | EKFTLSYLPDLTEEQLGKEVDYLLR | 5.2 | 2998.5537 |
| 126 | 4.56E+08 | DPLPPVSYLVDLTEEQLGKEVDYLLR | 3.8 | 3000.5693 |
