# Supplementary figures and images for: Evaluation of potential anti-metastatic and antioxidative abilities of natural peptides derived from Tecoma stans (L.) Juss. ex Kunth in A549 cells
Source: PeerJ. 2022 Jul 6;10:e13693. doi: 10.7717/peerj.13693 (PMC9270879; doi:10.7717/peerj.13693)

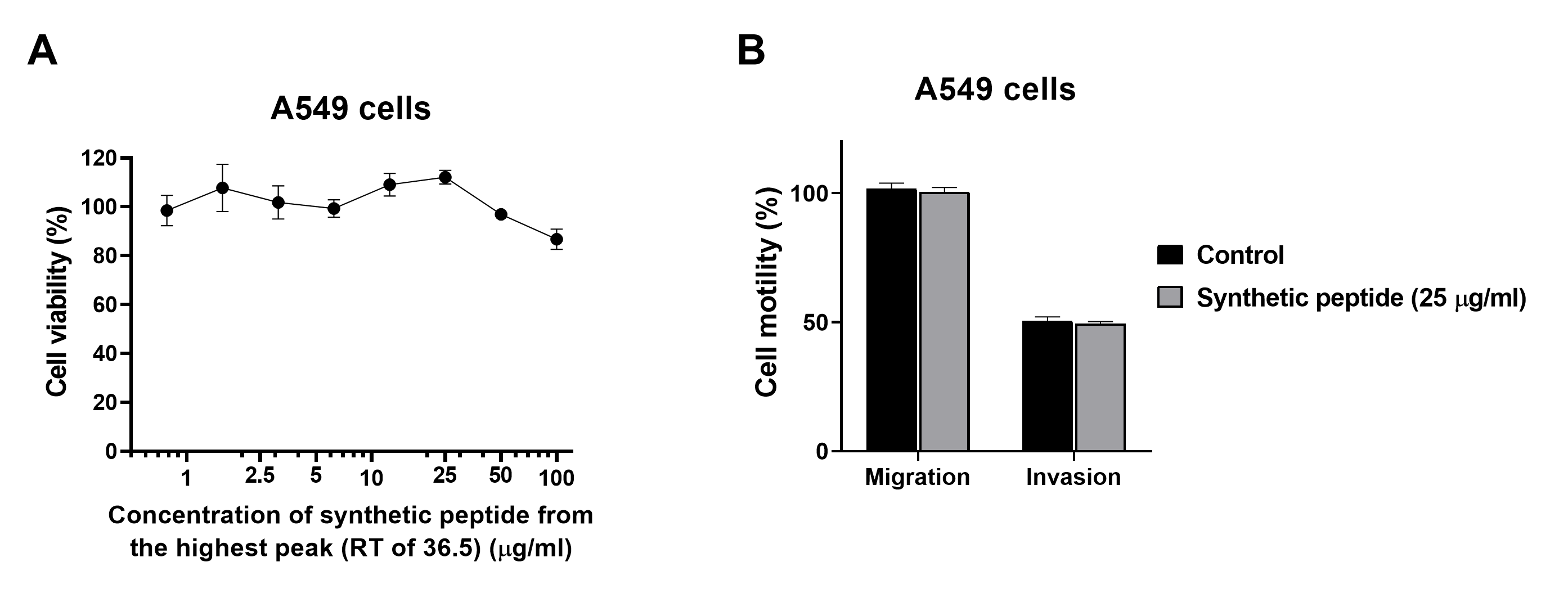

Supplement: Supplemental Information 2 — (A) Cell viability of A549 cells treated with various concentrations of the synthetic peptide from the highest peak (RT of 36.5 min). (B) Cell migration of A549 cells treated with 25 µg/ml synthetic peptide. [file peerj-10-13693-s002.png]

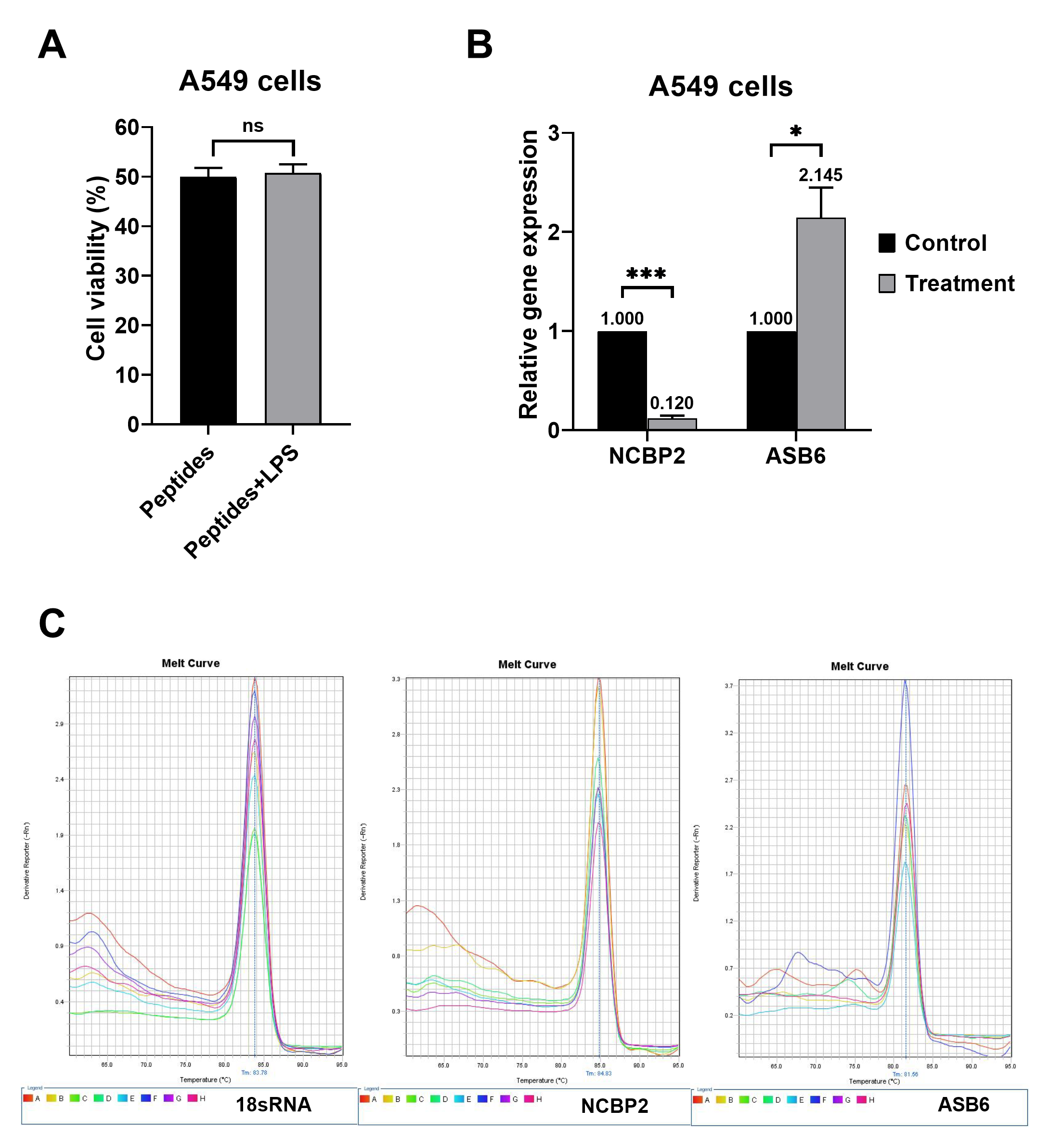

Supplement: Supplemental Information 3 — (A) Cell viability of A549 cells pretreated with 0.3321 ng/ml of natural peptides for 6 h, stimulated with or without 0.5 µg/ml LPS for 24 h. (B) Relative gene expressions of NCBP2 and ASB6 in A549 cells pretreated with or without 0.3321 ng/ml of natural peptides for 6 h, followed by 0.5 µg/ml LPS for 24 h. The expression of NCBP2 and ASB6 was normalized with that of 18S rRNA and shown as relative expression. The expression of NCBP2 and ASB6 in treated cells was relative to that obtained from the untreated control cells, which was arbitrarily set as 1. * and *** for p-values <0.05 and <0.001, respectively. (B) The melt curve analysis of 18S rRNA, NCBP2, and ASB6. [file peerj-10-13693-s003.png]

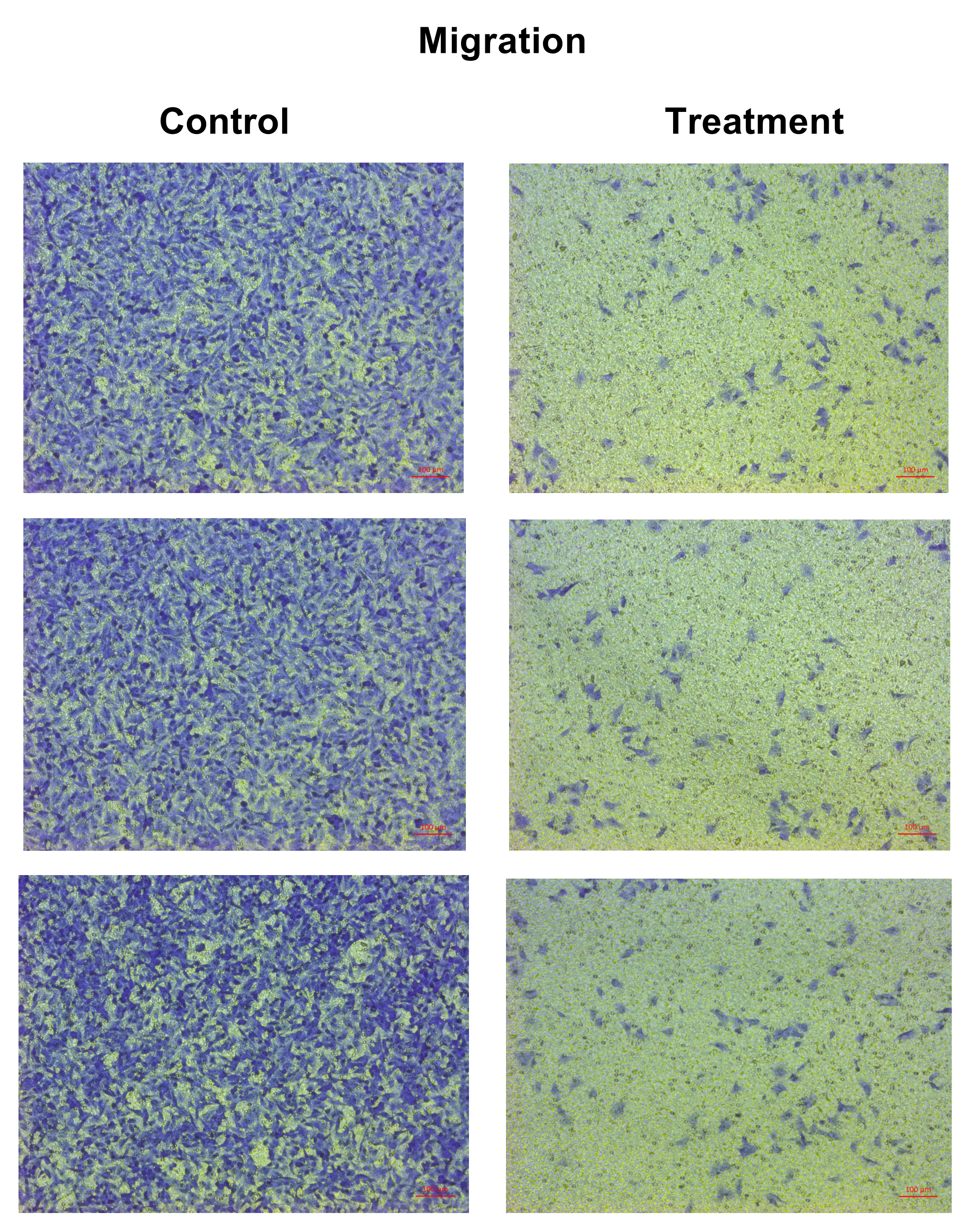

Supplement: Supplemental Information 7 [file peerj-10-13693-s007.png]

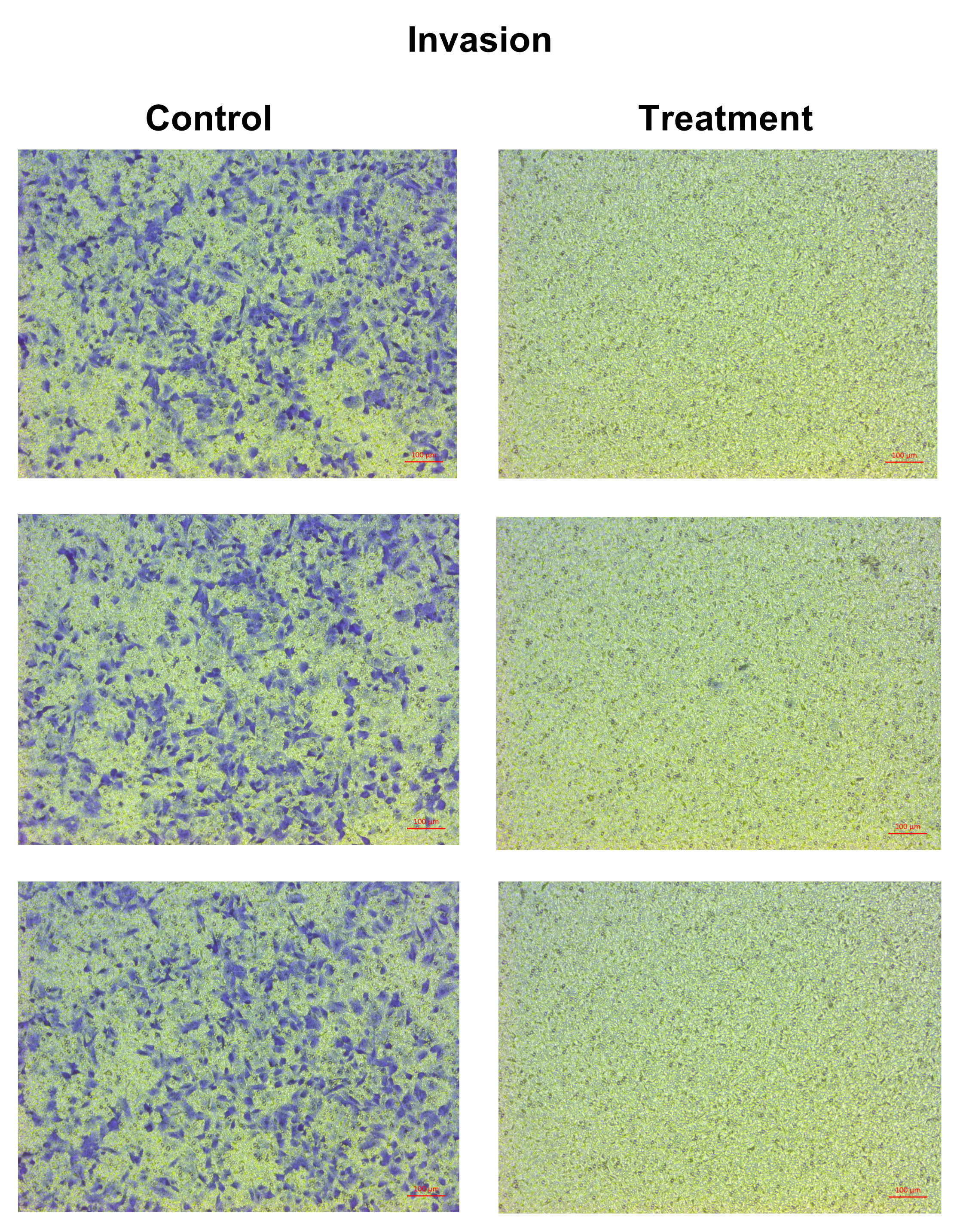

Supplement: Supplemental Information 8 [file peerj-10-13693-s008.png]
